# Association Between Use of Cannabis in Adolescence and Weight Change into Midlife

**DOI:** 10.1371/journal.pone.0168897

**Published:** 2017-01-06

**Authors:** Lexie Zhiyan Jin, Anna Rangan, Jesper Mehlsen, Lars Bo Andersen, Sofus C. Larsen, Berit L. Heitmann

**Affiliations:** 1 School of Molecular Bioscience, University of Sydney, Camperdown, New South Wales, Australia; 2 Coordinating Research Centre, Frederiksberg Hospital, The Capital Region, Frederiksberg, Denmark; 3 Exercise Epidemiology Unit, Department of Sports Science and Clinical Biomechanics, University of Southern Denmark, Odense, Denmark; 4 Research unit for Dietary Studies, the Parker Institute, Frederiksberg and Bispebjerg Hospitals, The Capital Region, Frederiksberg, Denmark; 5 The Institute of Public Health, Section for General Medicine, University of Copenhagen, Copenhagen, Denmark; 6 Boden Institute of Obesity, Nutrition Exercise & Eating Disorders, University of Sydney, Camperdown, New South Wales, Australia; 7 National Institute of Public Health, University of Southern Denmark, Odense, Denmark; University of Alabama at Birmingham, UNITED STATES

## Abstract

Cannabis use has been found to stimulate appetite and potentially promote weight gain via activation of the endocannabinoid system. Despite the fact that the onset of cannabis use is typically during adolescence, the association between adolescence cannabis use and long-term change in body weight is generally unknown. This study aims to examine the association between adolescence cannabis use and weight change to midlife, while accounting for the use of other substances. The study applied 20 to 22 years of follow-up data on 712 Danish adolescents aged between 15 and 19 years at baseline. Self-reported height and weight, cannabis, cigarette and alcohol use, socioeconomic status (SES) and physical activity levels were assessed in baseline surveys conducted in 1983 and 1985. The follow-up survey was conducted in 2005. In total 19.1% (n = 136) of adolescents reported having used/using cannabis. Weight gain between adolescence and midlife was not related to cannabis exposure during adolescence in either crude or adjusted models, and associations were not modified by baseline alcohol intake or smoking. However, cannabis use was significantly associated with cigarette smoking (p<0.001) and alcohol intake (p<0.001) and inversely associated with physical activity levels (p = 0.04). In conclusion, this study does not provide evidence of an association between adolescence cannabis use and weight change from adolescence to midlife.

## Introduction

Cannabis is widely used by younger age groups (15–34 years) [1, 2]. Cannabis use (both endogenous and exogenous cannabinoids) leads to the activation of the endocannabinoid system, which modulates the neuronal activity of other neurotransmitters through its action on the cannabinoid receptors in the brain, more specifically the cannabinoid receptor 1 (CB1) [3]. This activation stimulates appetite and food intake and hence may promote weight gain [4]. Despite of the fact that previous studies have found that up to 90% of tetrahydrocannabinol (THC, an active ingredient of cannabis) is excreted within 5 days of administration [5] the effects of THC on brain, especially the morphological changes happening in the CB1 receptor rich orbitofrontal cortex, seems substantial [6]. The orbitofrontal cortex has been found to be important in relation to termination of food intake, and the structure alterations that may occur in cannabis users, may have overconsumption of food as a long-term effect [7].

The use of cannabis based medicine (dronabino, nabilone and a cannabis extract [THC: cannabidiol = 1:1]) as an appetite stimulant has been legalized in some states in the United States (US) and some European countries to treat cancer and HIV related weight loss [8]. The ability of cannabis to reliably stimulate appetite has led to the development of cannabinoid receptor antagonists for the treatment of obesity. Four double-blind trials have shown that daily administration of Rimonabant, a CB1 receptor antagonist, significantly reduced weight in overweight or obese patients compared with placebo [9–12]. However, Rimonabant was also found to have psychogenic side effects such as increased anxiety, depression and suicidality and was removed from the market in Europe, and the application was withdrawn before the Food and Drug Administration approved it in the US [13].

In recent decades, the effects of cannabis use on body weight have been extensively studied [14–21]. Although, the onset of cannabis use is typically during adolescence, there has been little focus on potential effects on weight development of adolescent cannabis use. The adolescent period is a stage of high neuroplasticity in which the brain undergoes its final development and maturation [22]. An epigenetic effect of adolescent THC exposure has been discovered in animal studies that could impact on the expression of genes involved in brain development. This could ultimately result in alterations of adult brain functioning and behavior [23]. Hence, exposures to cannabis during developmental periods such as adolescence may have lead to body weight gain and may result in midlife overweight or obesity.

Although previous studies in humans have demonstrated an association between cannabis use and an increase in appetite and food intake [15, 21], large epidemiological studies have found conflicting results in relation to weight, with the majority indicating an inverse association with body weight (Table 1). One of these cross-sectional surveys conducted among 50,736 US adults aged 18 and over concluded that the prevalence of obesity was lower among cannabis users than among non-users [14]. Similar findings were reported by other cross-sectional studies; where current cannabis use was related to a lower Body Mass Index (BMI) [15–17] and an inverse relationship between BMI and cannabis use in the past 12 months was found for US women [18]. Conversely, a cross sectional nationally representative study in the US showed that frequent use of cannabis was associated with being overweight for 11–14 year old girls, but not in boys [19]. However, one recent longitudinal study among 5,141 adolescents, found that a consistent or increased use of marijuana from aged 12 to 18 years was associated with an increased risk of obesity in young adulthood [20]. On the other hand, another long term prospective study did not find a significant relationship between cannabis use and BMI over 15 years in young adults aged 18–30 years [21].

**Table 1:** Summary of the results from previous studies investigating the association between cannabis use and body weight.

Paper	Method/Study type	Population	Outcome	Conclusion
[14]	Cross-sectional	50,736 adults aged 18 years or older	Self-reported height and weight	Inverse association: lower obesity prevalence in cannabis users
[15]	Cross-sectional	10,623 adults aged 20–59 years	Measurement of height and weight were nots specified	Inverse association: current cannabis users had higher caloric intakes, but slightly lower BMI
[16]	Cross-sectional	6281 participants aged 20–59 years	Measured height and weight	Inverse association: current cannabis users had significantly lower BMI and waist circumferences
[17]	Cross-sectional	786 adults aged 18–74 years	Self-reported height and weight	Inverse association: cannabis use was statistically associated with lower BMI
[18]	Cross-sectional	297 morbid obesity females patients aged 16–79 years	Weight and height were collected from patient charts	Inverse association: higher use of cannabis in women with lower BMI
[19]	Cross-sectional	7825 adolescents aged 11–17 years	Self-reported height and weight	Direct association: overweight is associated with cannabis use in younger girls (aged 11–14), but not boys or girls aged 14–17 years
[20]	Longitudinal	5,141 adolescents aged from 12–18 years	Self-reported height and weight	Direct association: consistent or increased patterns of marijuana use in adolescence are associated with an increased risk of obesity
[21]	15 years longitudinal	3,617 young adults aged from 18–30 years	Measured height and weight	No association: no relationship between the use of cannabis and BMI

The aim of the present study was to examine the association between adolescence cannabis use and weight change from adolescence into midlife. The use of other substances such as smoking cigarettes and alcohol consumption were also examined as these are likely to modify the association between cannabis use and adiposity [24, 25].

## Method

### Data

This study was based on 20 to 22 years of follow-up among 3008 Danish teenagers aged between 15 and 19, who participated in two surveys conducted in either 1983 or 1985. Via a questionnaire on lifestyle and sports participation, subjects were asked questions on height and weight, socioeconomic status (SES), physical activity and substance use including cannabis, alcohol and tobacco in both the baseline and the follow-up study.

**Fig 1:**
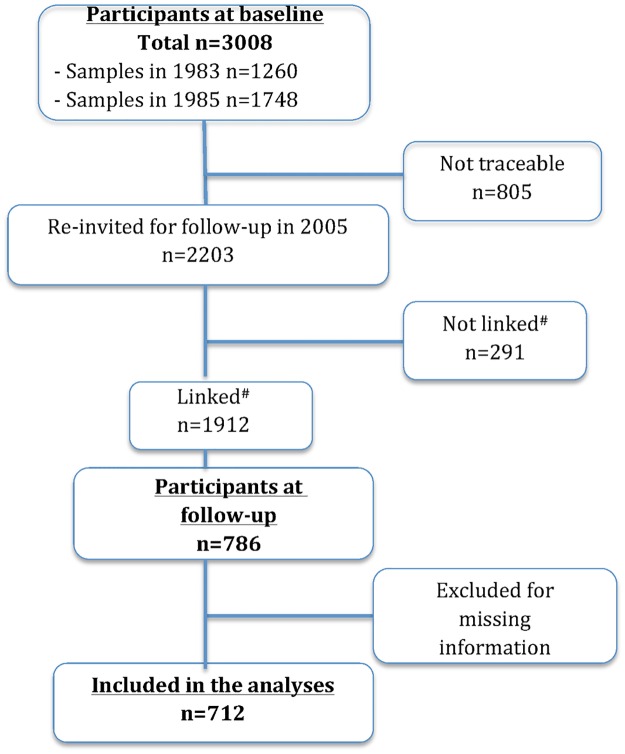
Study population flow chart. Fig 1 provides information on the participants flow between the baseline and follow-up surveys.

Of the 3008 participants, 805 could not be contacted at follow-up due to incomplete contact information, missing linkage information, a request not to be contacted for research purposes, left Denmark, or had death. Subjects that were traceable (2203 or 73%) were asked to fill out a mailed follow-up questionnaire. Of these, 786 (36%) returned the questionnaire (Fig 1). Dropout analysis revealed that age did not differ at follow-up between participants and non-participants (p = 0.25), but participants had slightly lower mean baseline BMI than non-participants (20.3 vs. 20.6 kg/m^2^; p = 0.004), and a higher proportion of participants than non-participants were in the highest SES class (scale 1) (33.9% vs. 26.7%; P = 0.004) [26]. After excluding participants with incomplete data on BMI information at baseline or follow-up, as well as on cannabis use, age, alcohol intake, smoking status, physical activity level, SES level, gender at baseline a total of 712 (32% of those traceable) subjects were eligible for inclusion in the present study Fig 1.

The study was conducted in accordance with the Helsinki Declaration. At the baseline data collection, there was no request for ethical approval, nor was there a consent procedure for minors. However, at the follow-up study in 2005, all participants provided written informed consent and approval was obtained from the Danish Data Protection Agency (Journal number: 2015-41-3940), as well as the local ethical committee (The scientific ethical committee for Copenhagen and Frederiksberg [KF 01-260/04]).

### Measures

#### Exposure

The use of cannabis was assessed with the question “Have you tried cannabis?” For this question, the participants were given six possible response categories: 1) No (80.9%); 2) Yes, but only once (5.3%); 3) Yes, a few times (7.0%); 4) Yes, but I have stopped (1.7%); 5) Yes, I still smoke once in a while (4.4%); 6) Yes, I smoke often (0.7%). Due to the small percentage of the participants who reported regular use of cannabis (0.7%) in comparison to those who reported no use (80.9%), we classified respondents who answered *no* as abstainers while the remaining were considered users (e.g. current or previous users of cannabis once or more). Supplementary analysis was also performed by grouping cannabis intake into three groups:abstainers (who answered *no* in the questionnaire), experimenters (who answered *Yes*, *but only once* and *Yes*, *a few times*) and frequent users (who answered *Yes*, *but stopped*, *Yes*, *and I still smoke occasionally* and *Yes*, *and I still smoke regularly*.) (S1 Table).

#### Outcome

BMI was calculated using self-reported height and weight for both baseline and follow-up surveys. Differences in BMI between baseline and follow-up were calculated as a continuous variable. BMI≥25.0kg/m^2^ was defined as overweight.

#### Covariates

Alcohol consumption was assessed by the question “How often do you drink different types of alcohol?” with three choices—beer, wine and spirit. The following assumptions were made to quantify the amount of alcohol consumption according to the possible answers to the question: 1) Never– 0 standard drink per week; 2) Very rarely– 0.08 (1/12) standard drink per week; 3) Once a month– 0.25 standard drink per week; 4) Twice per month– 0.5 standard drink per week; 5) Once a week– 1 standard drink per week; 6) 2–3 times per week—2.5 standards drink per week; 7) everyday– 7 standard drinks per week. All drinks were assumed to be standard drinks, and were calculated as units per week, and summed to create total alcohol consumption.

Smoking was assessed with the question “Do you smoke?” and responses categorised into smokers and non-smokers.

Participants were asked to report parental occupation and the highest education at the time when the participants were 10–12 years old. Those, with > 4yr of further education (master level), white-collar workers with > 50 subordinates, and self-employed with >20 employees were placed in SES group 1. Persons with short or medium further education (up to 4yr), white-collar workers with 11–50 subordinates (if no master level education), and self-employed with 6–20 employees (if no master level education) were placed in SES group 2. Persons with short education (up to 1yr), vocational education, white-collar workers with 1–10 subordinates (if no education corresponding to SES group 1 or 2), and self-employed with 0–5 employees (if no education corresponding to SES group 1 or 2) were placed in SES group 3. Skilled workers and white-collar workers with no subordinates (if no education corresponding to SES group 1, 2, or 3) were placed in SES group 4. Semiskilled and unskilled workers were placed in SES group 5 [27]. The two highest SES groups (1 and 2) were collapsed and labeled ‘high SES’, SES group 3 was labeled ‘medium SES’ and the two lowest SES groups (4 and 5) were collapsed and labeled ‘low SES’. SES at baseline was based on paternal SES. If no SES could be assigned to the father, maternal SES was used.

Subjects were asked for the different types of the sports they pursued at school and the number of hours per week spent on each sport. Leisure time physical activity was assessed by questionnaire, where participants filled in the different types of sports clubs they attended and the time they actively participated in each type of sport per week. Lastly, the type and the amount of time spend on activities outside of school and sports clubs were also assessed. Each sport and activity was translated into a metabolic equivalent (MET) scores [28]. Total energy expenditure from all activities was calculated for each participant.

### Statistical analysis

Descriptive analyses for the cohorts’ characteristics were conducted according to cannabis use, and tested using independent unpaired t-test for continuous variables and chi-square test for categorical variables. To estimate the association between cannabis use and changes in BMI, univariate generalized linear models were used, followed by stratification of the sample by gender and baseline smoking status. Crude estimation of associations was identified first, and potential covariates were adjusted for in subsequent models. Baseline covariates included smoking, alcohol intake, BMI, SES and physical activity levels. Effect modification was examined with respect to gender, age, BMI, smoking and alcohol use during adolescence by testing for the interactions with cannabis use in relation to adult BMI. As a sensitivity test, a logistic regression model with adult overweight (BMI ≥25.0kg/m^2^) as categorical outcome was fitted with the exposure to cannabis. Results were considered significant when p<0.05 (two-sided). SPSS Statistics version 21 was used for all analyses.

## Results

### Participant characteristics

Characteristics of the participants are given in Table 2. Of the 712 eligible participants, 136 (19.1%) reported use of cannabis during adolescence. Cannabis use at baseline was associated with cigarette smoking (p<0.001), and alcohol intake (p<0.001) irrespective of the type of alcohol. Cannabis use was inversely associated with baseline physical activity (p = 0.04). Gender, baseline BMI, and SES levels were not related to cannabis use.

**Table 2:** Participant characteristics according to cannabis use at baseline.

	Characteristics	Cannabis Abstainers	Cannabis Exposures	p-value^
		n = 576	n = 136	
**Mean ± SEM**	**Age baseline (years)**	16.9 ± 0.04	17.2 ± 0.09	0.002
	**BMI (kg/m**^**2**^**)** Baseline	20.3 ± 0.09	20.5 ± 0.17	0.42
	**BMI (kg/m**^**2**^**)** Follow-up	24.4 ± 0.16	24.4 ± 0.30	0.55
	**Total alcohol intake baseline** (drinks/week)	1.32 ± 0.06	2.60 ± 0.18	<0.001
	Beer	0.64± 0.04	1.44 ± 0.12	<0.001
	Wine	0.44 ±0.03	0.73 ± 0.08	0.001
	Spirit	0.31 ± 0.02	0.58 ± 0.06	<0.001
	**Physical Activity** (in MET score)	5.37 ± 0.30	4.00 ± 0.43	0.04
	**Gender (%)**	-	-	0.36
	Male	41.8	37.5	
	Female	58.2	62.5	
	**SES** (%)	-	-	0.19
	High	32.3	40.4	
	Medium	29.0	25.0	
	Low	38.7	34.6	
	**Smoking (%)**	-	-	<0.001
	Non-smoker	86.3	43.4	
	Smoker	13.7	56.6	

^ Independent t-test for continuous variables and chi-square tests for trend for categorical variables across cannabis use.

### Cannabis use in adolescence and subsequent changes in BMI

There were no significant differences in the changes in BMI from adolescence to midlife between cannabis abstainers and cannabis users in the crude model (Table 3), and results were essentially similar after adjusting for baseline gender, baseline BMI, age, SES, physical activity, smoking status and alcohol consumption. The changes in BMI were also similar for cannabis abstainers and cannabis users after stratifying the sample by alcohol intake (Table 4). When stratifying the sample by smoking status, those who did not smoke but used cannabis had the smallest numerical change in BMI of 3.5 kg/m^2^, while those who were both cigarette and cannabis free had the greatest numerical change in BMI of 4.1kg/m^2^ but these differences were not significant in any of the models (Table 5). There also were no differences in the odds ratios for overweight/obesity (BMI ≥ 25 kg/m^2^) between cannabis abstainers and users in the logistic regression analyses (results not shown). As a supplementary test, we grouped cannabis use into abstainers, experimenters and frequent users, and performed univariate and adjusted logistic regression analysis (S2 Table). Consistent with our previous analyses, no differences in weight gain between groups were detected. Finally, we compared the mean changes in BMI between cannabis abstainers at both baseline and follow-up, to those who reported using cannabis at both surveys (Table 6). Again, the results revealed no association between cannabis use and changes in BMI.

**Table 3:** Mean changes in BMI (95%CI) (kg/m2) between adolescence and midlife according to baseline cannabis use.

	Abstainers	Exposures	p-value
	(n = 576)	(n = 136)	
**Crude**	4.1 (3.8, 4.4)	3.7 (3.2, 4.3)	0.22
**Adjusted for SES baseline**	4.1 (3.8, 4.4)	3.7 (3.2, 4.3)	0.26
**Adjusted for gender**	4.1 (3.8, 4.4)	3.8 (3.2, 4.3)	0.28
**Adjusted for Physical Activity baseline**	4.1 (3.8, 4.4)	3.7 (3.1, 4.2)	0.17
**Adjusted for BMI baseline**	4.1 (3.8, 4.4)	3.7 (3.2, 4.3)	0.24
**Adjusted for baseline alcohol intake**	4.1 (3.8, 4.3)	3.8 (3.3, 4.4)	0.46
**Adjusted for baseline smoking status**	4.1 (3.8, 4.4)	3.7 (3.1, 4.3)	0.21
**Adjusted for baseline age**	4.1 (3.8, 4.4)	3.8 (3.2, 4.3)	0.36
**Adjusted for all**	4.1 (3.8, 4.3)	3.9 (3.3, 4.5)	0.55

**Table 4:** Mean changes in BMI (95%CI) (kg/m2) between adolescence and midlife in stratified model by baseline alcohol intake according to baseline cannabis use.

	Cannabis Use	Low Alcohol Intake (25^th^ Percentile)	Moderate Alcohol Intake	High Alcohol Intake (75^th^ Percentile)
**Crude**	**Abstainers**	4.3 (3.9, 4.8)	4.1 (3.7, 4.6)	3.7 (3.2, 4.2)
		n = 228	n = 195	n = 153
	**Exposures**	3.3(1.6, 5.0)	4.1 (3.2, 5.1)	3.6 (2.9, 4.3)
		n = 17	n = 39	n = 80
	**P-value**^$^	0.24	0.99	0.80
**Adjusted**^#^	**Abstainers**	4.3 (3.8, 4.8)	4.2 (3.8, 4.6)	3.7 (3.2, 4.2)
	**Exposures**	3.7 (1.9, 5.6)	3.8 (2.9, 4.9)	3.7 (2.9, 4.4)
	**P-value**^$^	0.57	0.54	0.96

^#^ Adjusted for gender, BMI, age, physical activity, smoking and SES status at baseline.

^$^ p-values for differences between abstainers and exposures.

**Table 5:** Means changes in BMI (95%CI) (kg/m2) between adolescence and midlife in stratified model by baseline smoking status according to baseline cannabis use.

	Cannabis Use	Smokers	Non-smokers
**Crude**	**Abstainers**	4.1 (3.4, 4.8)	4.1 (3.8, 4.4)
		n = 79	n = 497
	**Exposures**	3.9 (3.1, 4.6)	3.5 (2.7, 4.3)
		n = 77	n = 59
	**P-value**^$^	0.70	0.18
**Adjusted**^#^	**Abstainers**	4.1 (3.3, 4.8)	4.1 (3.8, 4.4)
	**Exposures**	3.9 (3.2, 4.7)	3.8 (2.9, 4.6)
	**P-value**^$^	0.81	0.52

^#^ Adjusted for gender, BMI, age, alcohol consumption, physical activity and SES status at baseline.

^$^ p-values for differences between abstainers and exposures.

**Table 6:** The mean changes in BMI (95%CI) (kg/m2) between adolescence and midlife according to cannabis abstinence or exposure at both baseline and follow-up.

Changes in BMI (95%CI)	Abstainer at baseline and Abstainer at follow-up	Exposure at baseline and Exposure at follow-up	P value for the differences in group mean
	n = 360	n = 124	
**Crude**	4.2 (3.8, 4.5)	3.9 (3.3, 4.5)	0.43
**Adjusted for confounders at baseline**^a^	4.2 (3.8, 4.6)	3.9 (3.2, 4.6)	0.51
**Adjusted for confounders at follow-up**^b^	4.1 (3.9, 4.3)	4.2 (3.8, 4.6)	0.64

^a^ Adjusted for gender, BMI, age, alcohol consumption, physical activity, smoking and SES status at baseline.

^b^ Adjusted for gender, BMI, age, alcohol consumption, physical activity, smoking and SES status at follow-up.

## Discussion

Our study suggests that use of cannabis in adolescence was not related to long-term weight change in midlife.

The lack of association between cannabis use in adolescence and development of obesity in midlife could be explained by two main perspectives; indirect social process as well as biological reactions. First, Marijuana use, especially heavy use, may indicate membership in a social group whose weight-related norms act to suppress weight. It has been reported that marijuana use might be linked to unhealthy weight loss practices in adolescence such as ≥ 24h fasting, use of diet pills use or laxative use/purging [29]. Moreover, there is a substantial possibility for confounding because of the long interval between adolescence and mid-life that may be stronger than potential programming effects from adolescent cannabis exposure, and may explain why we did not see significant associations with long-term development in weight gain.

Second, the potential cannabis effects on increase of appetite could be outweighed by a higher metabolic rate among cannabis users [30], and resulting in the lack of association between cannabis use and weight gain observed in the present study. A previous small placebo-controlled study showed that smoking cannabis caused a 28% increase in the metabolic rate [30]. A higher metabolic rate in cannabis users might also be the reason why those with cannabis exposures had a similar BMI as cannabis abstainers even if they had significantly lower physical activity levels. Tetrahydrocannabivarin (THCV), an active ingredient in cannabis, has been shown to produces weight loss, decreased body fat and serum leptin concentrations with increased energy expenditure properties in animals [31, 32]. Therefore, this anti-obesity activity of THCV could have diminished the appetite stimulating function of THC’s [8].

In our study, although there was no statistical significant difference in BMI between abstainers and users, adolescents who used cannabis were less physically active than those who did not use it. It is possible that cannabis users are less motivated for doing exercise. In support of this, a previous study found that smoking cannabis caused morphological changes in orbital frontal cortex [7] which is involved in motivation [33].

Smoking is an important confounder in the association between the cannabis use and weight change as smoking is related to both body weight status [34, 35] and to cannabis use. Smoking hashish is the most frequent form of cannabis consumption in Denmark, and hashish is frequently mixed with tobacco when used. However, results obtained after adjustment for smoking were essentially similar to those obtained from the crude analysis. Furthermore, cannabis users also had higher alcohol consumption. However, as with smoking, there was no evidence that our results were dependent on differences in alcohol intake between users and non-users of cannabis, or that the association between cannabis use and long-term weight change was modified by alcohol intake.

The present study advances the field in several ways. First, our study used longitudinal data focusing on adolescence cannabis use and weight changes, which is an advance since most previous studies were cross sectional [14–19]. Moreover, majority of previous studies did not consider simultaneous use of other substances such as tobacco or alcohol intake [16–18, 20]. However, the study results must also be interpreted with care due to some limitations. First, cannabis use and BMI data were self-reported and may be biased. People generally tend to underestimate the actual usage of drugs as well as underreport their body weight while over-reporting their height [36]. Such reporting bias would tend to mask a true *direct* relation linking cannabis use to long-term risk of overweight and obesity, while it would tend to inflate a true *inverse* relation between cannabis and long term risk of overweight and obesity. Second, effects of cannabis use may be dependent on duration of usage, and the possible answers related to cannabis use, lacked the desired amount of details such as duration of usage. This may have led to an attenuation of our results. Third, the dropout from baseline to follow-up was large. It has been reported that dropout rates are often higher in the lower SES groups, as well as in the obese/diseased individuals compared with higher SES groups and normal-weight people [37]. The large drop-out rate also resulted in small sample size in our study. In addition to the small sample size, the distributions between measurements groups of cannabis were skewed. Despite the lack of statistical association between cannabis use and obesity, the numerical data agrees with the results of two US national cross-sectional studies, indicating an inverse rather than a direct association between cannabis use and obesity [14, 15]. This was also the case in the present study, where mean baseline BMI was higher in the group that did not participate in the follow-up survey compared to those who participated. Therefore, the statistical association could have been biased due to greater drop-out in low SES and high BMI individuals. Lastly, information on dietary intake, which is one of the major factors affecting body weight, was not available and hence could not be adjusted for in the present analyses. Apart from these biological factors, also social factors may have indirectly affected the association between cannabis use and body weight. Although, SES was a covariate in the analyses, it is only one measure of social factors. Hence, residual confounding is likely.

## Conclusion

This study did not find support that adolescent use of cannabis was related to subsequent weight change into midlife.

## Supporting Information

S1 Table Frequency of baseline cannabis use—supplementary test. (DOCX)

S2 Table Mean changes in BMI (95%CI) according to baseline cannabis use—the supplementary test. (DOCX)
