# Supplementary material for: Association Between Use of Cannabis in Adolescence and Weight Change into Midlife
Source: PLoS One. 2017 Jan 6;12(1):e0168897. doi: 10.1371/journal.pone.0168897 (PMC5218547; doi:10.1371/journal.pone.0168897)
Supplement: S1 Table — (DOCX) [file pone.0168897.s001.docx]

**S1 Table.** Frequency of baseline cannabis use – supplementary test.

|  | Frequency | Percentage |
| --- | --- | --- |
| Abstainers^#^ | 576 | 80.9 |
| Experimenters^$^ | 88 | 12.4 |
| Frequent users^^^ | 48 | 6.7 |

^#^Abstainers - who answered ‘no’ in the questionnaire

^$^Experimenters – who answered ‘Yes, but only once’ and ‘Yes, a few times’

^^^Frequent users – who answered ‘Yes, but stopped’, ‘Yes, and I still smoke occasionally’ and ‘ Yes, and I still smoke regularly.’
