# Supplementary material for: Association Between Use of Cannabis in Adolescence and Weight Change into Midlife
Source: PLoS One. 2017 Jan 6;12(1):e0168897. doi: 10.1371/journal.pone.0168897 (PMC5218547; doi:10.1371/journal.pone.0168897)
Supplement: S2 Table — (DOCX) [file pone.0168897.s002.docx]

**S2 Table.** Mean changes in BMI (95%CI) according to baseline cannabis use – the supplementary test.

|  | **Abstainers**  **n = 611** | **Experimenters**  **n = 97** | **Frequent Users**  **n = 49** | **P-value for differences in the group mean** |
| --- | --- | --- | --- | --- |
| **Crude** | 4.1 (3.8, 4.4) | 3.3 (2.7, 4.0) | 4.4 (3.5, 5.3) | 0.09 |
| **Adjusted for alcohol intake** | 4.1 (3.8, 4.3) | 3.4 (2.7, 4.1) | 4.6 (3.7, 5.6) | 0.10 |
| **Adjusted for smoking status** | 4.1 (3.8, 4.4) | 3.3 (2.6, 4.0) | 4.4 (3.4, 5.4) | 0.09 |
| **Adjusted for SES^&^ baseline** | 4.1 (3.8, 4.4) | 3.4 (2.7, 4.1) | 4.4 (3.5, 5.3) | 0.11 |
| **Adjusted for age baseline** | 4.1 (3.8, 4.4) | 3.4 (2.7, 4.1) | 4.4 (3.5, 5.4) | 0.15 |
| **Adjusted for gender** | 4.1(3.8, 4.4) | 3.4 (2.7, 4.1) | 4.4 (3.5, 5.3) | 0.13 |
| **Adjusted for PA baseline** | 4.1(3.8, 4.4) | 3.3 (2.6, 4.0) | 4.4 (3.4, 5.3) | 0.08 |
| **Adjusted for BMI baseline** | 4.1 (3.8, 4.4) | 3.4 (2.7, 4.1) | 4.4 (3.5, 5.3) | 0.11 |
| **Adjusted for all** | 4.1 (3.8, 4.3) | 3.6 (2.9, 4.3) | 4.5 (3.5, 5.5) | 0.21 |

^&^ SES at baseline was based on parental SES. Each participant was assigned into five scales, 1 to 5, according to the scale described by the Danish National Centre for Social research. High SES: scale 1-2; Medium SES: scale 3 and Low SES: scale 4-5.
